# Nomogram for Predicting Cardiovascular Mortality in Incident Peritoneal Dialysis Patients: **An Observational Study**

**DOI:** 10.1038/s41598-017-14489-4

**Published:** 2017-10-24

**Authors:** Xi Xia, Chen Zhao, Qimei Luo, Qian Zhou, Zhenchuan Lin, Xiaobo Guo, Xueqin Wang, Jianxiong Lin, Xiao Yang, Xueqing Yu, Fengxian Huang

**Affiliations:** 1 The First Affiliated Hospital of Sun Yat-sen University, Department of Nephrology, Guangzhou, 510080 People’s Republic of China; 2 Ministry of Health and Guangdong Province, Key Laboratory of Nephrology, Guangzhou, 510080 People’s Republic of China; 3 Anhui Provincial Hospital Affiliated to Anhui Medical University, Department of Nephrology, Hefei, 230001 People’s Republic of China; 4 School of Mathematics & Computational Science, Sun Yat-sen University, Department of Statistical Science, Guangzhou, 510275 People’s Republic of China; 5 Sun Yat-Sen University, Southern China Research Center of Statistical Science, Guangzhou, 510275 People’s Republic of China; 6 Sun Yat-Sen University, Zhongshan School of Medicine, Guangzhou, 510080 People’s Republic of China

## Abstract

Cardiovascular mortality risk is high for peritoneal dialysis (PD) patients but it varies considerably among individuals. There is no clinical tool to predict cardiovascular mortality for PD patients yet. Therefore, we developed a cardiovascular mortality risk nomogram in a PD patient cohort. We derived and internally validated the nomogram in incident adult PD patients randomly assigned to a training (N = 918) or a validation (N = 460) dataset. The nomogram was built using the LASSO Cox regression model. Increasing age, history of cardiovascular disease or diabetes were consistent predictors of cardiovascular mortality. Low hemoglobin and serum albumin, high hypersensitive C-reactive protein and decreasing 24 hours urine output were identified as non-traditional cardiovascular risk predictors. In the validation dataset, the above nomogram performed good discrimination (1 year c-statistic = 0.83; 3 year c-statistic = 0.78) and calibration. This tool can classify patients between those at high risk of cardiovascular mortality (high-risk group) and those of low risk (low-risk group). Cardiovascular mortality was significantly different in the internal validation set of patients for the high-risk group compared to the low-risk group (HR 3.77, 2.14–6.64; *p* < 0.001). This novel nomogram can accurately predict cardiovascular mortality risk in incident PD patients.

## Introduction

Despite great improvements in the last decades, mortality risk in dialysis patients remains about 6.1 to 16 times greater than that of the general population^1,2^. Indeed, cardiovascular disease (CVD) is the leading cause of mortality in dialysis patients, accounting for approximately 40% of deaths^1,2^. However, cardiovascular mortality risk among individual dialysis patients varies considerably. Identification and risk stratification of dialysis individuals with cardiovascular mortality risk is an important issue in clinical practice and helps caregivers to appropriately inform patients and optimize individualized decision making.

In asymptomatic adults, the Framingham risk score has been one of the widely used tools to estimate individual risk of cardiovascular events and has been validated in racially diverse general populations^3^. Nevertheless, the Framingham risk score underpredictes cardiovascular risk in a predominantly stage 3 chronic kidney disease (CKD) population aged 45 to 74 years and the 10-year C-statistics assessing discrimination were 0.60 and 0.73 for men and women, respectively^4^. Moreover, a study of 201 hemodialysis patients aged 20–80 years old reports that high risk (>20% 10-year risk) categorized by Framingham risk score cannot predict cardiovascular mortality^5^.

In dialysis patients, no cardiovascular risk predictive instrument has been widely accepted currently and there is limited data on this issue yet. Some studies have found that scores of comorbidity, malnutrition-inflammation status, or vascular calcification, and models or scores with clinical and laboratory data can predict cardiovascular risk in dialysis patients^6–11^. However, previously developed risk scores could not be easily implemented in clinical practice for using some variables not routinely measured^6–9^ or time-consuming^6,7^. For example, coronary artery calcification score is assessed using computed tomography which are not routinely performed due to the exposure to X-ray and high cost^6^. Malnutrition-inflammation score contains 10 subjective and objective items and takes at least 15 minutes for a trained doctor^9^. Furthermore, generalizability of these scores may be limited by small sample sizes^6–9^ and subjectivity of evaluator-dependency^6–9^. In addition, the models or scores developed in hemodialysis patients may be not applicable to the PD patients because PD patients had less hemodynamic change and better preservation of residual renal function but greater loss of albumin^10,11^.

In this study, the objective was to develop an accurate but simple prediction tool for PD patients to estimate risk of cardiovascular mortality using only characteristics commonly available at the time of starting PD therapy.

## Results

### Characteristics of Study Participants

**Figure 1:**
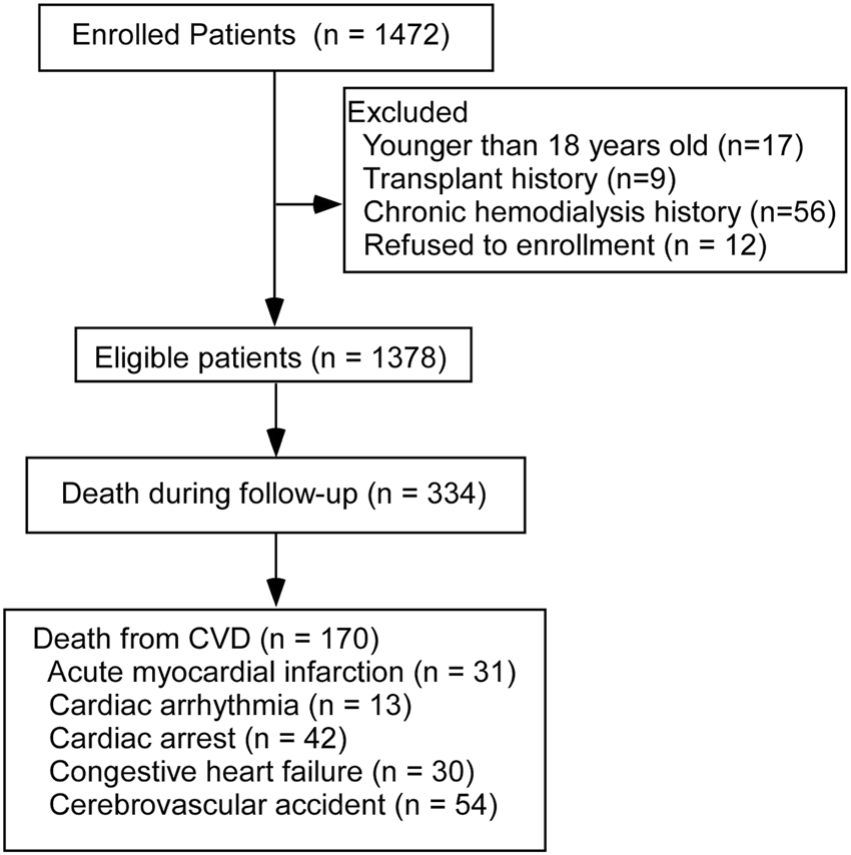
Enrollment and outcomes of the cohort. Abbreviations: CVD; cardiovascular disease.

Overall, 1378 eligible patients were included in the analysis and followed for a median duration of 39.7 months (Fig. 1). During the follow-up time, a total of 334 patients died, of which 170 (50.9%) deaths were attributed to CVD. Patients in the training and validation datasets were similar in demographic characteristics, comorbidities, laboratory data, use of medicine and outcomes (Table 1).

**Table 1:** Baseline Characteristics of the study populations and subpopulations.

Characteristics	Total (n = 1378)	Training dataset (n = 918)	Validation dataset (n = 460)	*P* value^a^
Demographics				
Age (years)	48.3 ± 15.6	48.4 ± 15.6	48.1 ± 15.5	0.78
No. of men	796 (57.8)	523 (57.0)	273 (59.3)	0.42
Body mass index (kg/m^2^)	21.6 ± 2.9	21.5 ± 3.0	21.7 ± 2.9	0.36
Smoking	296 (21.5)	198 (21.6)	98 (21.3)	0.95
Comorbid conditions, n (%)				
Diabetes	357 (25.9)	245 (26.7)	112 (24.3)	0.36
Hypertension	877 (63.6)	590 (64.3)	287 (62.4)	0.51
Cardiovascular disease	376 (27.3)	251 (27.3)	125 (27.2)	1.00
Systolic blood pressure (mmHg)	137.7 ± 19.8	137.5 ± 19.3	138.2 ± 20.9	0.52
Diastolic blood pressure (mmHg)	84.2 ± 14.1	84.2 ± 14.2	84.2 ± 13.9	0.95
Laboratory data				
Hemoglobin (g/L)	94.2 ± 18.9	94.6 ± 18.8	93.6 ± 19.0	0.38
Serum albumin (g/L)	36.2 ± 4.5	36.2 ± 4.5	36.0 ± 4.6	0.28
Albumin-corrected calcium (mmol/L)	2.3 ± 0.2	2.3 ± 0.2	2.3 ± 0.2	0.34
Serum phosphorus (mmol/L)	1.7 ± 0.5	1.7 ± 0.4	1.7 ± 0.5	0.08
Triglycerides (mmol/L)	1.4 [0.9]	1.4 [0.9]	1.4 [1.0]	0.60
HDL-C (mmol/L)	1.2 ± 0.3	1.2 ± 0.3	1.1 ± 0.4	0.18
LDL-C (mmol/L)	2.9 ± 0.8	2.9 ± 0.8	2.8 ± 0.9	0.60
Hs-CRP (g/mL)	3.0 [6.5]	3.0 [6.9]	2.9 [5.8]	0.87
Serum uric acid (μmol/L)	426.3 ± 92.6	427.2 ± 92.2	424.4 ± 93.4	0.60
Serum creatinine (μmol/L)	870.7 ± 301.1	868.7 ± 307.5	874.7 ± 288.3	0.72
Alkaline phosphatase (U/L)	65.0 [30.0]	66.0 [30.0]	63.0 [28.8]	0.10
iPTH (pg/ml)	276.0 [316.3]	283.1 [311.0]	250.0 [331.1]	0.42
Kt/V	2.4 ± 0.6	2.4 ± 0.6	2.4 ± 0.5	0.43
24 hours urine output	700 [700]	700 [650]	700 [700]	0.20
RKF (ml/min/1.73 m^2^)	3.6 ± 2.9	3.7 ± 2.9	3.6 ± 2.8	0.78
Medication				
ACEi/ARB	854 (62.0)	560 (61.0)	294 (63.9)	0.32
Follow-up time (months)	39.7 [38.9]	40.1 [38.8]	39.3 [38.3]	0.31
Death n (%)	334 (24.2)	226 (24.6)	108 (23.5)	0.69
Cardiovascular death n (%)	170 (12.3)	118 (12.9)	52 (11.3)	0.44

^a^For comparison between training dataset and validation dataset.

Abbreviations: ACEi, angiotensin-converting enzyme inhibitor; ARB, angiotensin receptor blocker; HDL-C, high density lipoprotein cholesterol; Hs-CRP, high-sensitivity C-reactive protein; iPTH, intact parathyroid hormone; LDL-C, low-density lipoprotein cholesterol; RKF, residual kidney function.

### Prediction Model of CVD mortality

**Figure 2:**
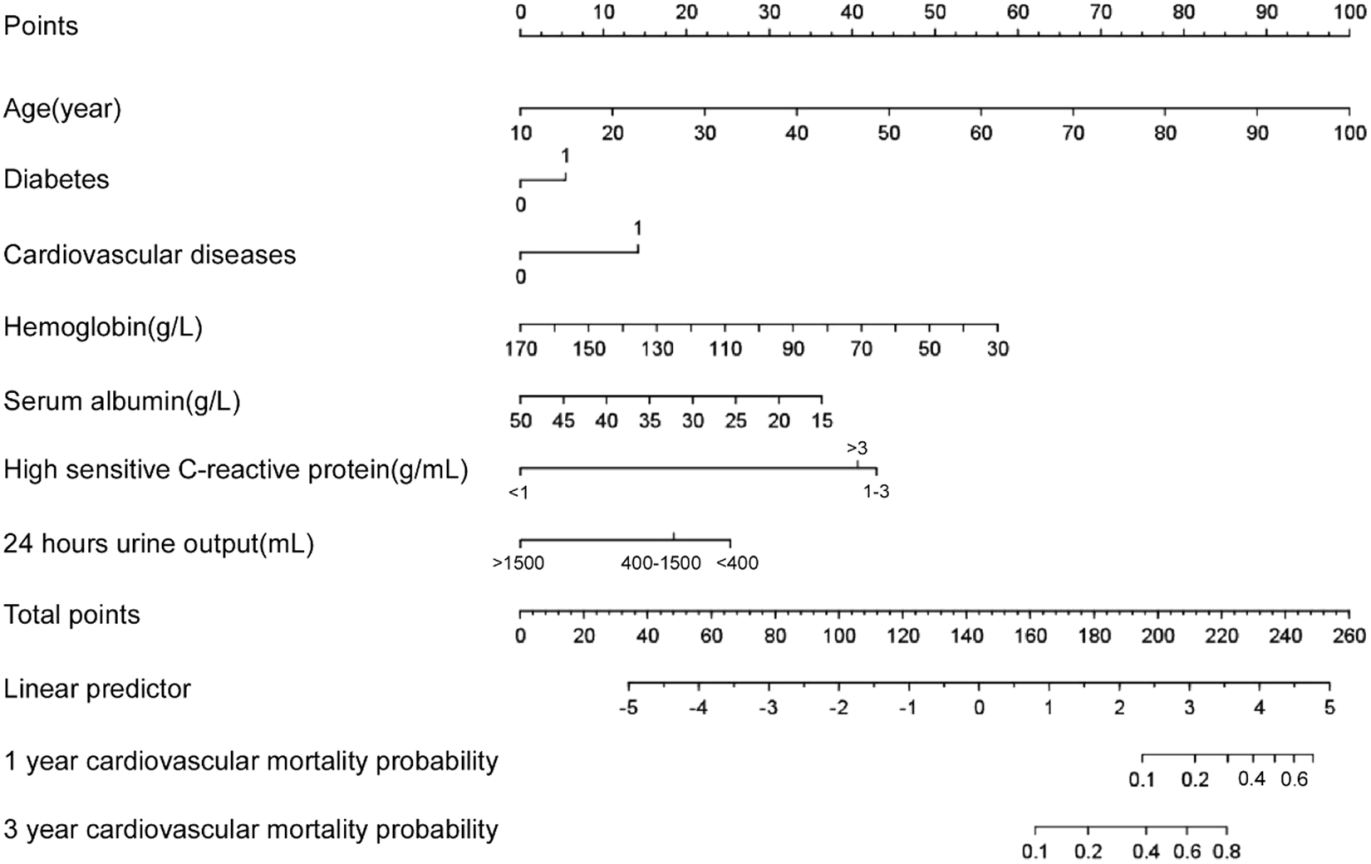
Nomogram to predict risk of cardiovascular mortality in peritoneal dialysis patients.

We used a LASSO Cox regression model to build the final prediction model, which selected seven variables from candidate variables in the training set: age, CVD, diabetes, albumin, hemoglobin, Hs-CRP, and 24-hours urine output (Table 2 and Supplemental Fig. 1). The linear predictors (Cox model coefficients) from the LASSO Cox regression model were used to develop the nomogram to predict cardiovascular mortality in PD patients (Fig. 2).

**Table 2:** Multivariate Cox Regression Model on cardiovascular Mortality in the Training Dataset.

Variable	Coefficient	Hazard Ratio (95% CI)	p-value
Age (per 1 year older)	0.0505208	1.05 (1.03–1.07)	<0.001
Cardiovascular disease (yes *vs*. no)	0.6451847	1.91 (1.29–2.82)	0.001
Diabetes mellitus (yes *vs*. no)	0.2472196	1.28 (0.86–1.90)	0.22
Albumin (per 1 g/L higher)	−0.0471919	0.95 (0.91–0.99)	0.046
Hemoglobin (per 1 g/L higher)	−0.0186977	0.98 (0.97–0.99)	0.002
Hs-CRP, g/mL			
<1		Reference	
1–3	1.9562888	7.07 (2.12–23.65)	0.001
>3	1.8543571	6.39 (1.98–20.56)	0.002
24-hours urine output, ml			
>1500		Reference	
400–1500	0.8396937	2.32 (0.72–7.42)	0.16
<400	1.1506428	3.16 (0.97–10.33)	0.056

Abbreviations: Hs-CRP, high-sensitivity C-reactive protein.

### Prediction Nomogram Performance in the Training Dataset

**Figure 3:**
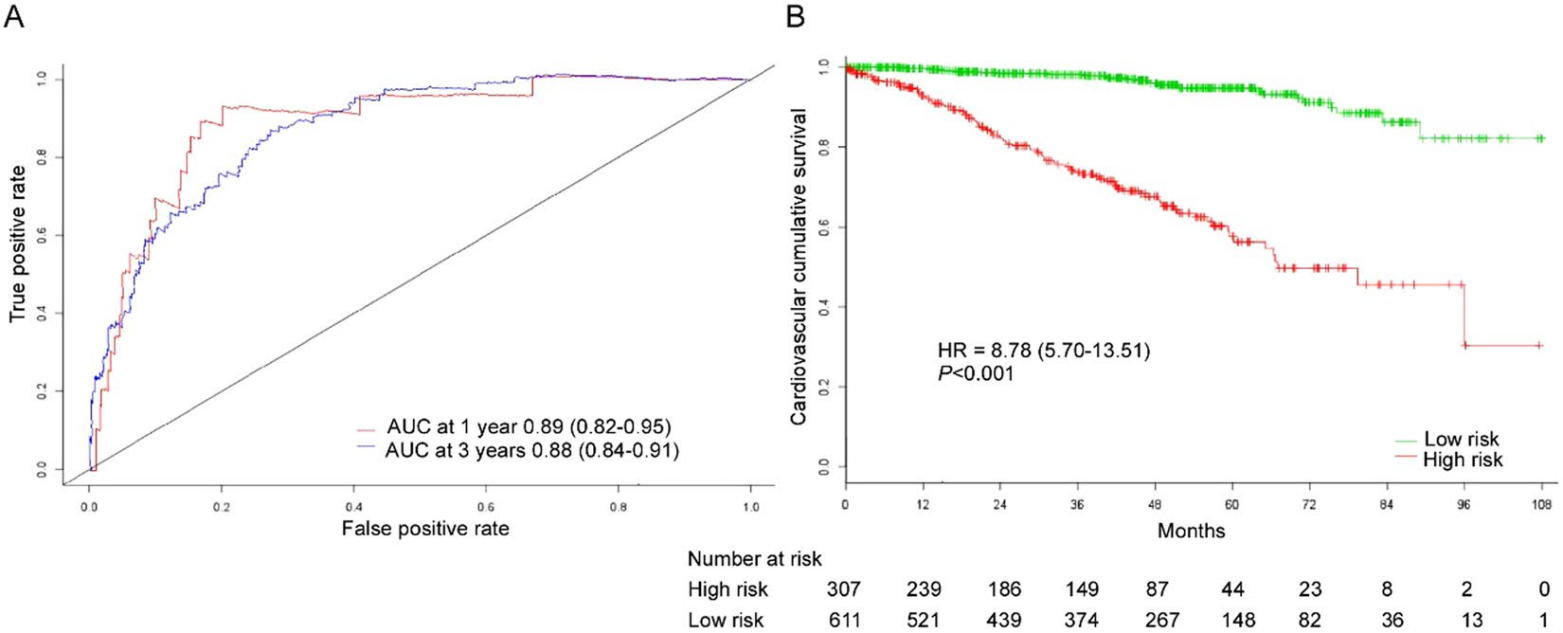
(**A**) Time-dependent ROC curves and (**B**) Kaplan-Meier survival curves in the training sets on the basis of the nomogram. Abbreviations: ROC; receiver operator characteristic, AUC; area under the curve.

**Figure 4:**
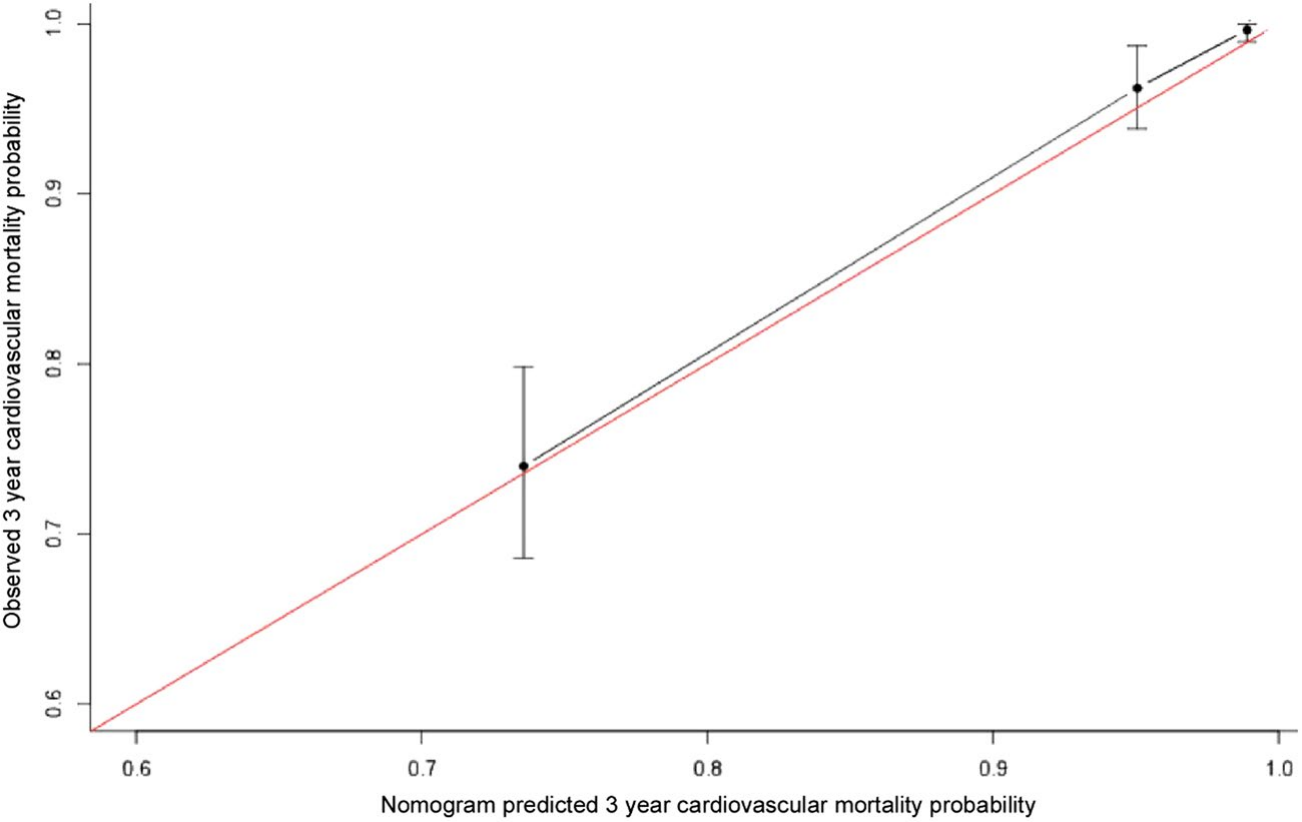
Plots depict the calibration of the nomogram in terms of agreement between predicted and observed 3-year outcomes in the training sets. Model performance is shown by the plot, relative to the 45-degree line, which represents perfect prediction.

The nomogram had good performance in cardiovascular mortality prediction, with time-dependent AUC of 0.89 (95% CI, 0.82–0.95) and 0.88 (95% CI, 0.84–0.91) at 1 and 3 years, respectively (Fig. 3A). Two risk categories were defined on the basis of the nomogram and Kaplan-Meier curves showed that patients in the high risk group had significantly higher cumulative rate of cardiovascular mortality (HR 8.78, 95% CI 5.70–13.51; *p* < 0.001; Fig. 3B). The calibration plot showed that the predicted probability of 3 years cardiovascular mortality by the nomogram and the actual observed cardiovascular mortality was relatively well matched (Fig. 4).

### Prediction Nomogram Performance in the Validation Dataset

**Figure 5:**
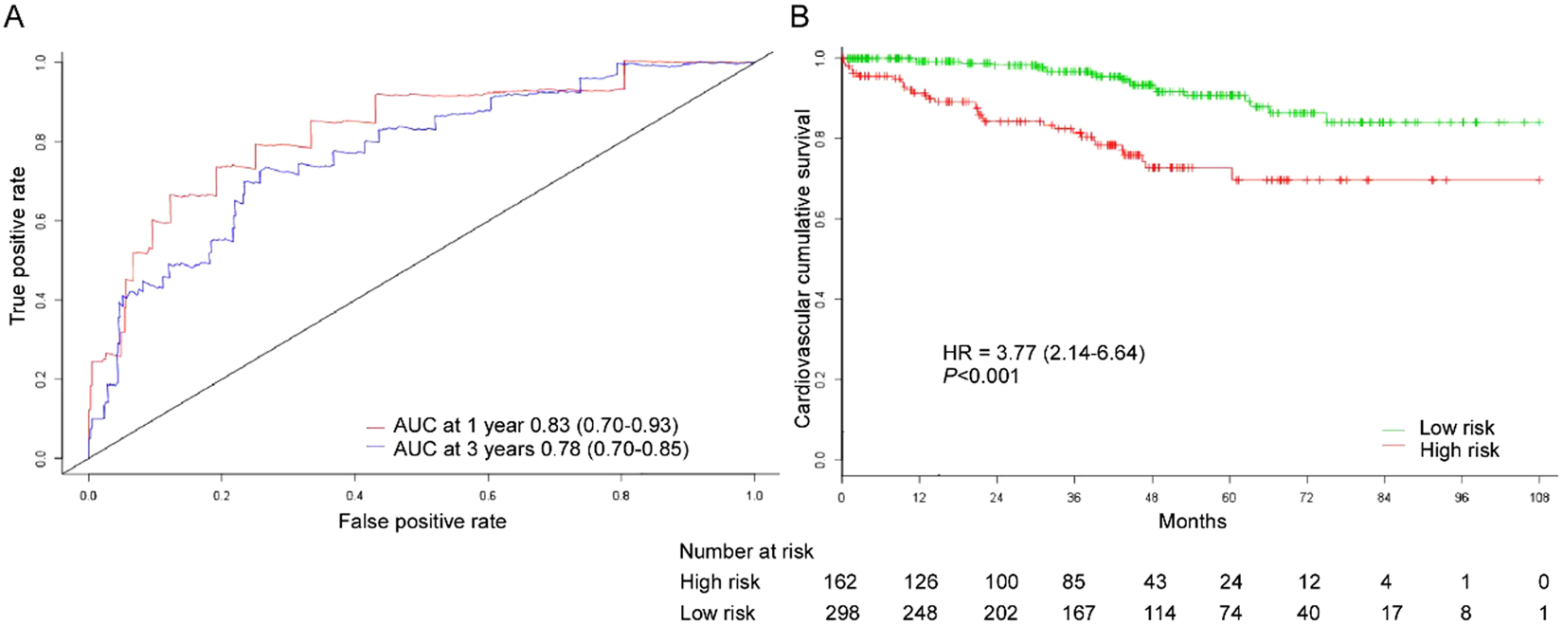
(**A**) Time-dependent ROC curves and (**B**) Kaplan-Meier survival curves in the internal testing sets on the basis of the nomogram. Abbreviations: ROC; receiver operator characteristic, AUC; area under the curve.

**Figure 6:**
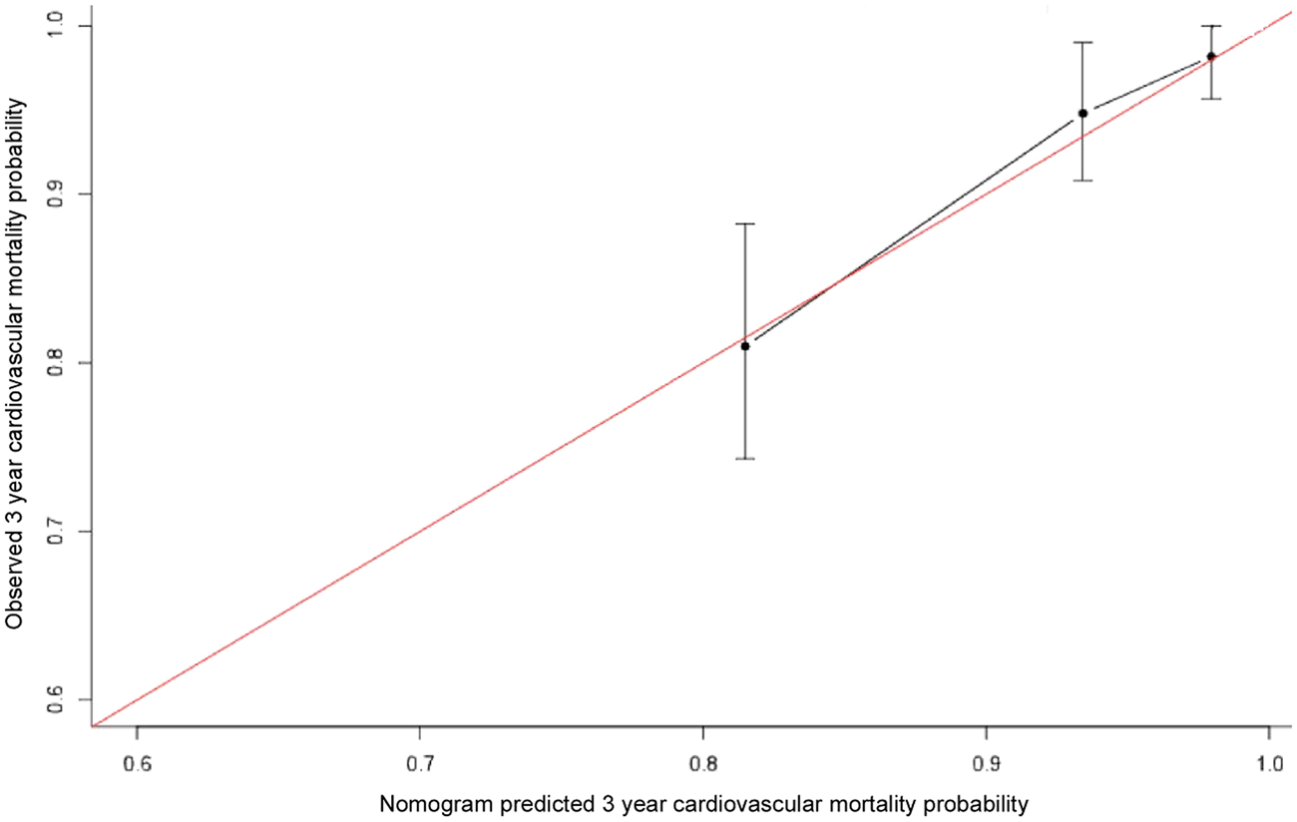
Plots depict the calibration of the nomogram in terms of agreement between predicted and observed 3-year outcomes in the internal testing sets. Model performance is shown by the plot, relative to the 45-degree line, which represents perfect prediction.

The accuracy of the nomogram at predicting cardiovascular mortality in the validation set was high as well, with time-dependent AUC of 0.83 (95% CI, 0.70–0.93) and 0.78 (95% CI, 0.70–0.85) at 1 and 3 years, respectively (Fig. 5A). The nomogram could also accurately classify patients into low-risk and high-risk subgroups and the cumulative incidence of cardiovascular death was greater in the high risk group (HR 3.77, 95% CI 2.14–6.64; *p* < 0.001; Fig. 5B). In the internal validation cohort, the calibration plot for the probability of 3 years cardiovascular mortality showed a well agreement between prediction by the nomogram and the actual observation (Fig. 6).

## Discussion

This study developed and validated a novel prediction instrument for cardiovascular mortality risk among incident PD patients using seven easily available baseline variables, which included traditional cardiovascular risk factors and dialysis-specific factors. The prediction nomogram achieved sufficient accuracy and well discrimination.

Cardiovascular risk prediction tools were commonly applied in asymptomatic adults, which had improved patient outcomes with individualized risk prediction and interventions^12,13^. However, the applicability of cardiovascular risk prediction tools developed in asymptomatic adults was limited in CKD patients and clinically useful models for predicting cardiovascular risk in CKD patients were lacking^4,14^. This study developed a cardiovascular mortality prediction tool, which allowed early identification of patients at high risk of cardiovascular mortality. So, treatment decisions will be better informed and early interventions will benefit high risk patients. This nomogram suggested treatment of anemia, hypoalbuminemia, and inflammation and preserving residual urine output may be the key points to reduce cardiovascular risk for PD patients. Furthermore, it may also serve as a useful tool for the optimal selection of patients in clinical trials.

Many previous studies including ours had reported several all-cause mortality predictive models for dialysis patients^15–17^. Predictive models for cardiovascular mortality are more helpful for cause-specific intervention. There has been limited data in the development of prediction tool for cardiovascular risk among dialysis patients. Vascular calcification score based on plain radiographic films and coronary artery calcification score based on computed tomography were suggested as independent predictors of cardiovascular mortality in dialysis patients^8,9^. These radiographic exams was not routinely performed due to exposure to X-ray and observer-dependency limited standardized scoring and generalization. While the variables in our predictive nomogram were all routinely collected in clinical practice and the simple and standardized nomogram had good reproducibility. Some studies showed that malnutrition-inflammation score (MIS) or malnutrition inflammation depression arteriosclerosis (MIDA) score in dialysis patients can predict cardiovascular death^6,7^. However, these 2 studies only had small number of patients (81–141 participants) with limited events (8–26 deaths) and the scoring systems were complex and time consuming. Shastri *et al*. developed a risk model to predict sudden cardiac death only while the primary outcome of our study is cardiovascular mortality^10^. Anker *et al*. developed a risk-score for 2-year cardiovascular mortality in a Fresenius Medical Care-based hemodialysis patient cohort (AROii)^11^. Age, CVD history, diabetes, hemoglobin, albumin and C-reactive protein were found as common predictors in both our studies. But our data did not find blood pressure and serum creatinine to be cardiovascular mortality predictors in PD patients while 24-hours urine output was found to be a predictor of cardiovascular mortality in PD patients. These results suggests the existence of different cardiovascular risk factors between PD and hemodialysis population. Therefore, the risk-score based on data generated from a single commercial hemodialysis provider may be less generalizable to PD patients. The vascular calcification score (AUC = 0.72), Shastri’s model (c-statistic = 0.75) and Anker’s model (c-statistic = 0.72–0.74) performed fair in predicting CVD mortality for dialysis patients^8,10,11^. Compared with these models, our nomogram performed good discrimination (1 year c-statistic = 0.83; 3 year c-statistic = 0.78).

Our analysis has a few strengths. First, the nomogram is practical because all the variables included are easily and routinely collected in clinical care and it takes less than 5 minutes to calculate individual cardiovascular mortality risk. Second, the data was collected on a relatively large population of incident PD cohort with regular follow-up from the starting of PD therapy and the rate of loss of follow-up was very low (3%). Third, both short term (1 year cardiovascular mortality) and long term outcomes (3 years cardiovascular mortality) were judged.

There are a few limitations of the current study as well. First, other markers of CVD, including N-terminal pro brain natriuretic peptide, troponin T, left ventricular function assessed using echocardiography, have been shown to predict CVD risk in dialysis patients and they are not incorporated in the present risk nomogram^18–20^. But these tests are not cheap and they are not routinely performed for all PD patients in our center at present. Second, the severity of diabetes and CVD comorbidity were not considered. However, there were no standard method to estimate the severity of these comorbidity diseases so far. Third, the changes of variables over time and treatments during the follow-up were not included. Yet, the aim of this nomogram was to aid risk stratification at the start of PD for early inform and intervention when only baseline data was available. Forth, although the robustness of our nomogram was examined extensively with internally validation using bootstrap testing, the generalizability was uncertain for other PD populations outside China. It needs to be externally evaluated in wider PD populations.

In conclusion, this study developed a novel nomogram with good accuracy to aid physicians in estimating the risk of 1 to 3 years cardiovascular mortality in patients starting PD therapy. With an estimate of individual risk, physicians and patients can make more informed decisions on life-style and medical interventions. This nomogram requires external validation and future studies are needed to determine whether individual targeting treatments based on this nomogram will reduce cardiovascular mortality.

## Methods

### Study population

This study enrolled consecutive incident adult PD patients without a history of renal transplantation and/or chronic hemodialysis (>3 months on hemodialysis), from The First Affiliated Hospital, Sun Yat-sen University in China from January 1, 2006, to December 31, 2011. Patients refused to give written consents were excluded. Eligible participants provided informed consent. This study adhered to the Declaration of Helsinki. The study protocol was approved by the Clinical Research Ethics Committee of The First Affiliated Hospital, Sun Yat-sen University.

### Candidate Predictors

Clinical and laboratory data was obtained using standardized forms. Candidate variables included demographic variables, including age, gender, and smoking status; physical examination variables, including blood pressure, body weight with dry abdomen, and height; comorbid conditions, including diabetes, hypertension, CVD; and laboratory variables from serum, urine and PD fluids were collected within 3 months after the initial of PD therapy. Body mass index was calculated according to the weight and height. Diabetes was defined by use of hypoglycemic medications and/or history of clinical diagnosis. Hypertension was based on prescription of antihypertensive drugs or 2 separate blood pressure measurements ≥140/90 mm Hg. History of angina pectoris, myocardial infarction, angioplasty, coronary artery bypass, heart failure, or stroke taken from clinical records was considered as CVD. Laboratory data including hemoglobin, albumin, creatinine, serum uric acid, albumin-corrected calcium, phosphorus, total triglycerides, high-density lipoprotein cholesterol, low-density lipoprotein cholesterol, intact parathyroid hormone (iPTH), alkaline phosphatase, and high-sensitivity C-reactive protein (Hs-CRP), were measured in The First Affiliated Hospital, Sun Yat-sen University. Residual kidney function (RKF) and total Kt/V were calculated using PD Adequest software 2.0 (Baxter Healthcare Ltd). RKF, in milliliters per minute per 1.73 m^2^, was estimated from mean values of creatinine clearance and urea clearance and adjusted for body surface area by the Gehan and George equation^21^.

### Outcome

The outcome of interest was cardiovascular mortality. Cardiovascular mortality was defined as death due to acute myocardial infarction, congestive heart failure, cardiac arrhythmia, cardiac arrest due to other or unknown causes, cerebrovascular accident. CVD death were confirmed according to death certificates if death occurred in any hospital. If death happened outside a hospital, CVD death classifications required independent audits by three experts in our center after a comprehensive consideration of the patient’s medical records and descriptions of caregivers. In total, 235 of 334 (70.4%) patients died in a hospital. All patients were followed up until death, undergoing a renal transplant, transfer to hemodialysis therapy, transfer of care from our center, or end of the study on December 31, 2014.

### Statistical analysis

The final dataset was randomly divided in a training dataset (2/3 of the original cohort, n = 918) and a validation dataset (1/3 of the original cohort, n = 460). Continuous variables were presented as mean ± SD or median (interquartile range) and compared using an ANOVA or Kruskal-Wallis test as appropriate. While, categorical variables were given as proportions and compared using a χ^2^ test. The Hs-CRP, 24-hours urine output, iPTH, triglycerides, and alkaline phosphatase were transformed into categorical variables based on routine cutoff points in clinical practice due to its skewed distribution. The remainder of the variables were evaluated as linear predictors. All variables had less than 15% missing values and most of them had less than 5% missing values (Supplemental Table 1). All missing data was imputed using the missForest method, which was a non-parametric method coping with different types of variables simultaneously^22^.

The proportional hazards assumption was verified by examination of scaled Schoenfeld residual plots. LASSO has been broadly applied to the Cox proportional hazard regression model for survival analysis^23^. LASSO Cox regression model was used to determine variable selection and constructed a model for predicting cardiovascular mortality in the training set. The baseline hazard function and coefficients from the training model were fixed and applied to the validation data set. Cox regression coefficients were used to generate the nomogram. We used time-dependent receiver operating characteristic (ROC) analysis and the area under the curve (AUC) at different cutoff times to measure predictive accuracy of the nomogram^24^. By using recursive partitioning tree analysis to generate the optimum cutoff point^25^, patients were categorized as ‘low’ or ‘high’ risk group. The Kaplan-Meier curves were plotted for these two risk groups and the log-rank test was used to compare survival curves. Bootstraps with 100 resamples were used for drawing calibration plots and comparing the observed with predicted risk of CVD mortality. All statistical tests were done with R software version 3.0.1. Statistical significance was set at 0.05 for two-tailed.

### Data Availability

The datasets generated during and/or analysed during the current study are available from the corresponding author on reasonable request.

## Electronic supplementary material

Supplementary Information

## References

[1] de Jager DJ (2009). Cardiovascular and noncardiovascular mortality among patients starting dialysis. JAMA..

[2] Saran R (2017). US Renal Data System 2016 Annual Data Report: epidemiology of kidney disease in the United States. Am J Kidney Dis..

[3] D’Agostino RB, Grundy S, Sullivan LM, Wilson P (2001). Validation of the Framingham coronary heart disease prediction scores: results of a multiple ethnic groups investigation. JAMA..

[4] Weiner DE (2007). The Framingham predictive instrument in chronic kidney disease. J Am Coll Cardiol..

[5] Huang JC (2013). Performance of the Framingham risk score in patients receiving hemodialysis. Nephrology..

[6] Ho LC, Wang HH, Chiang CK, Hung KY, Wu KD (2010). Malnutrition-inflammation score independently determined cardiovascular and infection risk in peritoneal dialysis patients. Blood Purif..

[7] Choi MJ (2012). The malnutrition-inflammation-depression-arteriosclerosis complex is associated with an increased risk of cardiovascular disease and all-cause death in chronic hemodialysis patients. Nephron. Clinical practice..

[8] Adragao T (2004). A simple vascular calcification score predicts cardiovascular risk in haemodialysis patients. Nephrol Dial Transplant..

[9] Xie Q (2016). Coronary artery calcification score as a predictor of all-cause mortality and cardiovascular outcome in peritoneal dialysis patients. Perit Dial Int..

[10] Shastri S (2012). Predictors of sudden cardiac death: a competing risk approach in the hemodialysis study. Clin J Am Soc Nephrol..

[11] Anker SD (2016). Development and validation of cardiovascular risk scores for haemodialysis patients. Int J Cardiol..

[12] Greenland P (2010). 2010 ACCF/AHA guideline for assessment of cardiovascular risk in asymptomatic adults: a report of the American College of Cardiology Foundation/American Heart Association Task Force on Practice Guidelines. Circulation.

[13] Batsis JA, Lopez-Jimenez F (2010). Cardiovascular risk assessment–from individual risk prediction to estimation of global risk and change in risk in the population. BMC Med..

[14] Tangri N (2013). Risk prediction models for patients with chronic kidney disease: a systematic review. Ann Intern Med..

[15] Zhao C (2014). Risk score to predict mortality in continuous ambulatory peritoneal dialysis patients. Eur J Clin Invest..

[16] Wagner M (2011). Predicting mortality in incident dialysis patients: an analysis of the United Kingdom Renal Registry. Am J Kidney Dis..

[17] Floege J (2015). Development and validation of a predictive mortality risk score from a European hemodialysis cohort. Kidney Int..

[18] Wang AY (2007). N-terminal pro-brain natriuretic peptide: an independent risk predictor of cardiovascular congestion, mortality, and adverse cardiovascular outcomes in chronic peritoneal dialysis patients. J Am Soc Nephrol..

[19] Khan NA, Hemmelgarn BR, Tonelli M, Thompson CR, Levin A (2005). Prognostic value of troponin T and I among asymptomatic patients with end-stage renal disease: a meta-analysis. Circulation..

[20] Zoccali C (2004). Left ventricular mass monitoring in the follow-up of dialysis patients: prognostic value of left ventricular hypertrophy progression. Kidney Int..

[21] Gehan EA, George SL (1970). Estimation of human body surface area from height and weight. Cancer chemotherapy reports..

[22] Stekhoven DJ, Buhlmann P (2012). Missforest–non-parametric missing value imputation for mixed-type data. Bioinformatics (Oxford, England)..

[23] Tibshirani R (1997). The lasso method for variable selection in the cox model. Statistics in medicine..

[24] Heagerty PJ, Lumley T, Pepe MS (2000). Time-dependent ROC curves for censored survival data and a diagnostic marker. Biometrics..

[25] Strobl C, Malley J, Tutz G (2009). An introduction to recursive partitioning: rationale, application, and characteristics of classification and regression trees, bagging, and random forests. Psychological methods..

