# Supplementary material for: Nomogram for Predicting Cardiovascular Mortality in Incident Peritoneal Dialysis Patients: An Observational Study
Source: Sci Rep. 2017 Oct 24;7:13889. doi: 10.1038/s41598-017-14489-4 (PMC5654762; doi:10.1038/s41598-017-14489-4)
Supplement: Supplementary file 1 — Supplementary Information [file 41598_2017_14489_MOESM1_ESM.pdf]

Nomogram for Predicting Cardiovascular Mortality in Incident Peritoneal Dialysis Patients: An Observational Study.

Xi Xia[1,2,+], Chen Zhao[1,2,3,+], Qimei Luo[1,2,+], Qian Zhou[1,2], Zhenchuan Lin[1,2], Xiaobo Guo[4,5], Xueqin Wang [4,5,6], Jianxiong Lin[1, 2], Xiao Yang [1, 2], Xueqing Yu[1, 2], Fengxian Huang[1, 2,*]

Supplemental material

Figure legends

Supplemental Figure 1 LASSO coefficient profiles of the baseline characteristics. A vertical line is drawn at the value chosen by 10-fold cross-validation.

Supplemental Figure 1

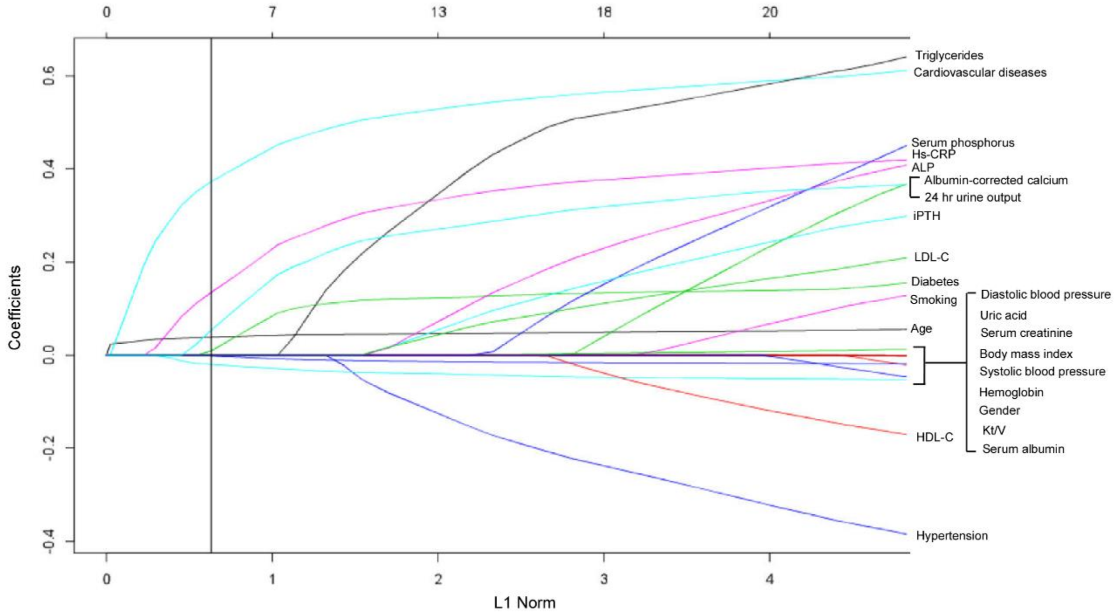

Supplemental Table 1 The number of missing values of baseline variables in the study populations.

Variables	Missing values n (%)
Demographics	
Age	0
Gender	0
Body mass index	104 (7.5)
Smoking status	42 (3.0)
Comorbid conditions	
Diabetes	0
Hypertension	0
Cardiovascular disease	0
Systolic blood pressure	15 (1.1)
Diastolic blood pressure	15 (1.1)
Laboratory data	
Hemoglobin	1 (0.1)
Serum albumin	0
Serum calcium	3 (0.2)
Serum phosphorus	4 (0.3)
Triglycerides	14 (1.0)
HDL-C	14 (1.0)
LDL-C	14 (1.0)

Hs-CRP	174 (12.6)
Serum uric acid	3 (0.2)
Serum creatinine	0
iPTH	28 (2.0)
Alkaline phosphatase	53 (3.8)
RKF	198 (14.4)
Kt/V	163 (11.8)
24-hours urine output	21 (1.5)
ACEi/ARB	0

Abbreviations: ACEi, angiotensin-converting enzyme inhibitor; ARB, angiotensin receptor blocker; HDL-C, high density lipoprotein cholesterol; Hs-CRP, high-sensitivity C-reactive protein; iPTH, intact parathyroid hormone; LDL-C, low-density lipoprotein cholesterol; RKF, residual kidney function.
